# Assessment of the Efficacy of Deep Brain Stimulation (DBS) in Managing Drug-Resistant Epilepsy (DRE): A Systematic Review and Meta-Analysis of Randomized Controlled Trials (RCTs)

**DOI:** 10.7759/cureus.71348

**Published:** 2024-10-13

**Authors:** Elshymaa E Raslan, Sultan F Al-Hawas, Moaath M Alghamdi, Abdalrhman S Alblwan, Anas Alhomaidhi, Abdulaziz A Alruwaili, Rabah Warar, Atheer S Alhwaiti, Rakan M Alsubhi, Wafa F Al-harbi, Majd Nouh Alasmari

**Affiliations:** 1 Surgery, King Khalid Hospital, Tabuk, SAU; 2 Neurosurgery, King Faisal Specialist Hospital & Research Centre, Riyadh, SAU; 3 Medicine and Surgery, Ibn Sina National College, Jeddah, SAU; 4 Medicine, Jouf University, Sakaka, SAU; 5 Medicine, King Saud Bin Abdulaziz University for Health Sciences, Riyadh, SAU; 6 Medicine, University of Tabuk, Tabuk, SAU; 7 Medicine and Surgery, Alfaisal University College of Medicine, Riyadh, SAU; 8 Medical Physics, Faculty of Medicine, University of Tabuk, Tabuk, SAU; 9 Medicine, Ibn Sina National College, Jeddah, SAU; 10 Medicine, Unaizah College of Medicine, AlQassim, SAU; 11 Medicine, King Khalid University, Abha, SAU

**Keywords:** deep brain stimulation, drug-resistant epilepsy, meta-analysis, responder rate, seizure reduction

## Abstract

Epilepsy is a chronic neurological disorder affecting millions of people around the world. Even though the majority of patients gain seizure control with antiseizure medications (ASMs), many subjects may have drug-resistant epilepsy (DRE). Deep brain stimulation (DBS) is a promising alternative, showing effectiveness in reducing seizures for some patients. This systematic review and meta-analysis aim to evaluate DBS’s efficacy in DRE. A comprehensive search in PubMed, CENTRAL, Medline, Ovid, and Scopus was performed up to August 31, 2024, using the terms ‘Drug-resistant Epilepsy’ AND ‘Deep Brain Stimulation’. Two independent researchers screened titles, abstracts, and full texts. The data extracted included study details, sample size, age at surgery, seizure duration, follow-up duration, seizure reduction (SR), responder rate (RR), and adverse events. Quality assessment was conducted using the Risk of Bias 2 (ROB2) tool (https://methods.cochrane.org/bias/resources/rob-2-revised-cochrane-risk-bias-tool-randomized-trials), and data analysis was performed using Jamovi software (https://www.jamovi.org). The search yielded 707 studies that were initially screened. Out of them, 29 articles were retrieved for full-text screening, and 5 randomized controlled trials (RCTs) were included in the review and meta-analysis. The meta-analysis showed that the pooled effect size for SR was 0.51 (95% CI, 0.35-0.68; P < 0.001), and the pooled effect size for RR was 0.54 (95% CI, 0.35-0.74; P < 0.001), demonstrating significant improvements in seizure control. The pooled effect size for adverse events was 0.21 (95% CI, 0.08-0.34; P = 0.001). The risk-of-bias assessment revealed a low risk of randomization for most studies. However, concerns were noted in areas such as deviations from the intended intervention and missing outcome data. In conclusion, DBS is a viable intervention for DRE, with significant reductions in seizure frequency and a favorable safety profile. However, the variability in efficacy and RRs across studies underscores the need for continued research to refine patient selection criteria, optimize stimulation parameters, and explore the differential effects of targeting various thalamic nuclei.

## Introduction and background

Epilepsy is a chronic neurological disorder characterized by recurrent seizures affecting approximately 50 million people around the world. Even though the majority of patients gain seizure control with antiseizure medications (ASMs), about 30% of subjects have drug-resistant epilepsy (DRE) [1]. DRE was defined as the inability to attain sustained seizure freedom after at least two selected and tolerated ASMs [2]. The condition presents important challenges, given the impairment not only in patients' quality of life but also in psychosocial well-being and cognitive function. It also presents a higher risk of sudden unexpected death in epilepsy (SUDEP) and other comorbidities [3]. In such cases, alternative therapeutic strategies to pharmacological intervention are needed when patients cannot respond to traditional ASMs.

Over the years, DBS has long been considered one of the promising treatments among patients with DRE, and this is more evident where surgical intervention is contraindicated or with multi-focal or generalized seizure types [4]. DBS includes implanting electrodes in specific brain regions to deliver electrical stimulation. This intervention modulates dysfunctional neural circuits and reduces seizure activity. Specifically, the anterior nucleus of the thalamus (ANT) and the centromedian nucleus of the thalamus (CM) have been widely studied as target regions due to their function in thalamocortical circuits implicated in epilepsy [5].

Recent randomized controlled trials (RCTs) have highlighted that stimulating the anterior thalamic nucleus (ANT-DBS) can significantly lower seizure frequency and severity when compared to a placebo or sham stimulation [6]. Similarly, research into centromedian thalamic stimulation (CM-DBS) has demonstrated positive results for patients dealing with generalized and multifocal seizures [7].

However, despite its efficacy, the response to DBS is highly variable, as some patients experience significant seizure reduction (SR) and others show minimal or no benefit. This variability indicates that there is a need for a better understanding of the mechanisms underlying DBS and the identification of biomarkers that could predict treatment outcomes [8]. Recent studies have suggested that differences in functional connectivity profiles within the brain may predict patient response to ANT-DBS, providing a biomarker for guiding patient selection and treatment optimization. Functional connectivity analysis using neuroimaging techniques like functional MRI can reveal distinct patterns of brain network organization that correlate with favorable DBS outcomes, allowing clinicians to tailor therapies based on individual patient profiles [5].

In addition, while DBS has shown to be well-tolerated, device-related complications, such as infections, lead migration, and hardware malfunctions, have been reported. Moreover, the adverse effects of stimulation, such as paresthesia, cognitive disturbances, and mood changes, must be weighed against the potential benefits and considered cautiously [9,10].

While much evidence is available to support DBS in epilepsy patients, the variability in study design, patient population, and stimulation parameters contributed to heterogeneity in results reported across clinical trials. Thus, a systematic review and meta-analysis would be required to sufficiently provide an overview of the efficacy and safety of DBS management in DRE patients.

The present systematic review and meta-analysis was performed to analyze data from RCTs on the effectiveness of DBS for DRE, addressing SR, responder rate (RR), and adverse events (AEs) as main outcomes. Based on the evidence synthesis from several studies, this review will be more valid and fill some gaps regarding the therapeutic effect of DBS to help in clinical decision-making in the management of drug-resistant epilepsy. These results may provide a basis for future clinical practice, selection of patients for treatments, and direction in research, finally improving outcomes in patients and advancing the frontiers of epilepsy treatment.

## Review

Methods

Methodology

The study was conducted in alignment with the guidelines outlined in the Cochrane Handbook for Systematic Reviews of Interventions, version 6, and the findings were reported following the Preferred Reporting Items for Systematic Reviews and Meta-Analyses (PRISMA) standards [11].

Eligibility Criteria

Inclusion criteria: The inclusion criteria for the study were: randomized controlled trials (RCTs) involving participants aged 18 years or older and diagnosed with drug-resistant epilepsy (DRE). Eligible studies had to provide detailed information on changes in seizure frequency, specifically focusing on seizure reduction (SR) and responder rate (RR). Additionally, only studies published in English were considered for inclusion.

Exclusion criteria: The exclusion criteria were as follows: studies that reported only qualitative data, as well as letters, reviews, editorials, observational studies, retrospective studies, and abstracts.

Search Strategy

An online search was conducted using five databases: PubMed, CENTRAL, Medline, Ovid, and Scopus to identify relevant studies published up to August 31, 2024, using subject words combined with free words. The terms were ‘Drug-resistant Epilepsy’ AND ‘Deep Brain Stimulation’. Aiming for a thorough exploration, examination of all likely applicable studies won't have geographical, racial, or age constraints. Further, detailed proofreading of references listed in retrieved articles and re-marks were identified to ensure comprehensive coverage and limit any oversights.

Selection of Studies

The processes of online search, screening the titles and abstracts, as well as revising the full text of relevant articles were conducted by two independent researchers. Any disagreements were resolved by consensus.

Data Extraction

The following data were extracted from the included studies: the author, publication year, target area, sample size of DRE patients, age at surgery, seizure duration, follow-up duration, seizure reduction (SR), response rate (RR) - defined as a seizure reduction rate of greater than 50% - and adverse events. SR was determined by comparing the seizure frequency at the final follow-up to that at baseline. The full text of the included articles was screened independently by two authors. Variables were calculated from the original data when the information was not clearly stated. Disagreements between reviewers were resolved by consensus.

Measured Outcomes

Primary outcome: The SR and RR were the measured primary outcomes.

Secondary outcome: The incidence of AE was the secondary outcome.

Assessment of the Quality of the Included Studies

We assessed the risk of bias (ROB) using the Risk of Bias 2 (ROB2) tool (https://methods.cochrane.org/bias/resources/rob-2-revised-cochrane-risk-bias-tool-randomized-trials) for randomized clinical trials (RCTs) [12], as all included studies were found to be RCTs. The ROB2 tool comprises five domains: randomization, deviations from the assigned treatment, missing data, measurement of the outcome, and selective reporting of the outcomes and results. Moreover, the overall ROB is assessed by selecting the highest level of ROB out of the five domains. The Robvis tool was used to visualize the figures [13].

Data Synthesis

All data analyses were performed using Jamovi software [14]. The primary outcomes were SR and RR. Heterogeneity between included studies was calculated using the Q test and I² statistic. A restricted maximum likelihood model was used when high heterogeneity was detected by the Q test. The mean SR and standard deviation (SD) of several studies were estimated based on data provided for the median and interquartile range (IQR). Since the number of studies was fewer than 10, publication bias was not examined.

Results

Characteristics of the Included Studies

**Figure 1:**
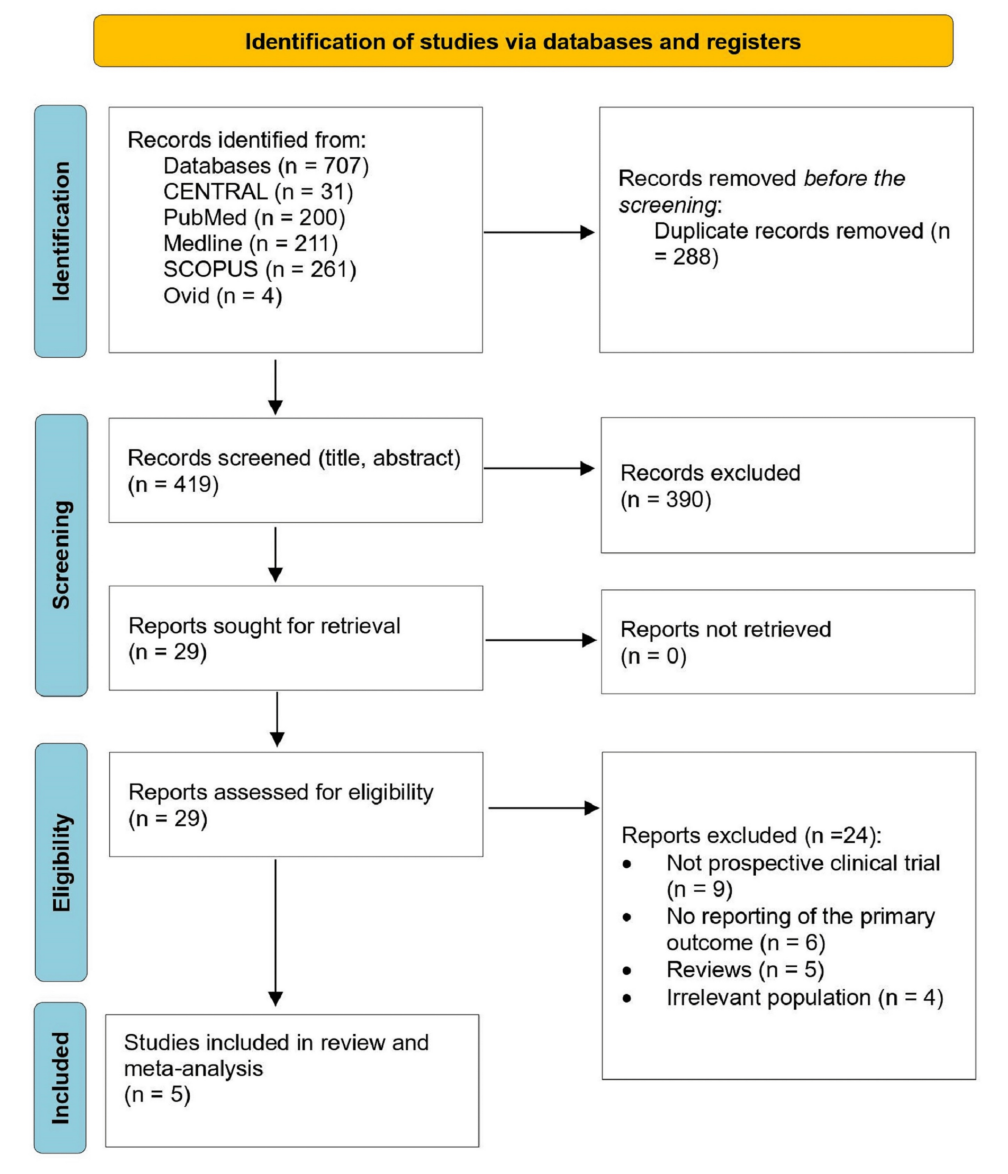
PRISMA flowchart selection process PRISMA: Preferred Reporting Items for Systematic Reviews and Meta-Analyses

The process of screening and selecting the included studies was based on Preferred Reporting Items for Systematic Reviews and Meta-Analyses (PRISMA) criteria (Figure 1). The retrieving process yielded 707 studies. A total of 288 studies were excluded as duplicates. The remaining 419 studies were screened for their titles and abstracts. Out of them, 390 were excluded. The remaining 29 records were reviewed for full text. During the exclusion process based on eligibility criteria, nine studies were excluded, as they were not prospective RCTs, 6 studies due to incomplete reporting of primary outcomes, 5 were reviews, and 4 had inappropriate populations. Finally, five articles were included for the review and meta-analysis (Table 1) [15-19].

**Table 1:** Characteristics of the included studies RS: seizure reduction; RR: responder rate; AE: adverse event; ANT: anterior nucleus of the thalamus; CM: Centromedian nucleus of the thalamus.

Study ID	Sample size	Age (mean ±SD)	DBS target	Duration (years)	Follow-up (months)	seizure reduction (SR)	responder rate (RR)	Adverse event
Dalic LJ,et al 2022 [15]	20	25±6.3	CM	21.7±7.4	86.5	0.54±0.31	0.40	0.35
Salanova V et al, 2021 [16]	73	37.1±11.8	ANT	22.5±13.9	120	0.75	0.74	0.13
Herrman H et al, 2019 [17]	18	NA	ANT	24	12	0.23±0.27	0.22	0.17
Salanova V et al. 2015 [18]	75	36.1	ANT	22.3	NA	0.49	0.68	NA
Fisher R et al, 2010 [19]	110	36.1±11.2	ANT	22.3±13.3	36±14.4	0.56	0.67	NA

Table 1 shows the characteristics of included DBS studies. A total of 296 adult DRE patients were included in the final statistical analysis. The number of patients varied from 18 to 110 in these studies. The mean age of the participants ranged between 25 and 37 years. DBS targeted the anterior nucleus of the thalamus (ANT) in all studies [16-19], except for Dalic LJ, et al [15], which targeted the centromedian nucleus of the thalamus (CM). All included articles were RCTs.

Meta-Analysis

**Figure 2:**
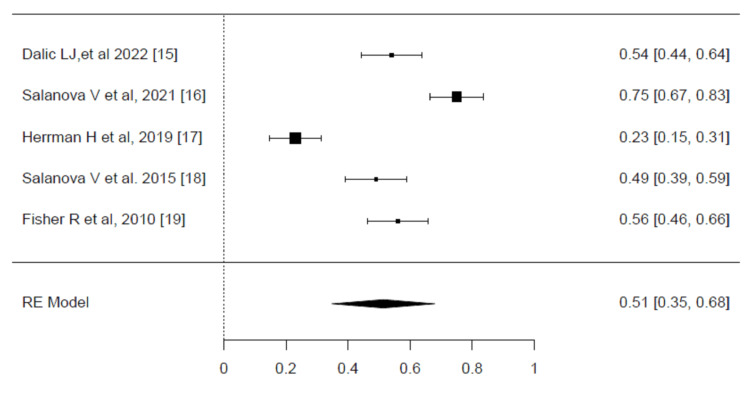
Meta-analysis of the seizure reduction (SR) Forest plot showing the pooled effect sizes for seizure reduction (SR) among patients treated with deep brain stimulation (DBS). The pooled effect size was 0.51 (95% CI, 0.35–0.68), indicating a moderate effect. Significant heterogeneity was detected (I² = 93.88%, p < 0.001), and the restricted maximum likelihood model was used for the analysis. Source: [15-19]

Seizure reduction (SR): The SR results of deep brain stimulation (DBS) are illustrated in the forest plots (Figure 2). A significant level of heterogeneity was observed across the included trials, as indicated by an I² value of 93.88% (p <0.001). Due to this high heterogeneity, the restricted maximum likelihood (REML) model was employed. The pooled effect size for SR among patients treated with DBS was found to be statistically significant, with an effect size of 0.51 (95% CI, 0.35-0.68, p <0.001). This result indicates that DBS has a moderate but consistent impact on reducing seizure frequency in patients, with the confidence interval reflecting the precision of the estimate [15-19].

**Figure 3:**
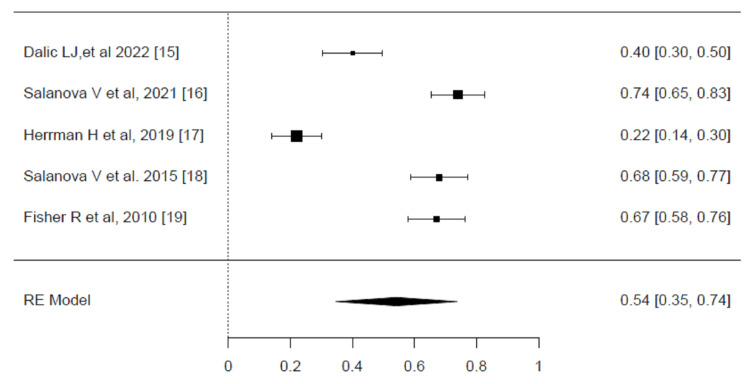
Meta-analysis of responder rate (RR) Forest plot showing the pooled effect sizes for responder rate (RR) among patients treated with deep brain stimulation (DBS). The pooled effect size was 0.54 (95% CI, 0.35–0.74), indicating a significant treatment effect. Significant heterogeneity was detected (I² = 95.87%, p < 0.001), and the restricted maximum likelihood model was used for the analysis. Source: [15-19]

Responder rate (RR): The responder rate (RR) results for DBS are presented in the forest plots (Figure 3). A high degree of heterogeneity was detected among the included trials, as indicated by an I² value of 95.87% (P <0.001). Given this significant heterogeneity, the restricted maximum likelihood (REML) model was employed to account for these variations and provide a more reliable pooled estimate of the treatment effect. The pooled effect size for RR among patients receiving DBS was statistically significant, with an effect size of 0.54 (95% CI, 0.35-0.74, P <0.001). This result indicates that over half of the patients treated with DBS experienced a clinically meaningful reduction in seizure frequency, as reflected by the responder rate. These results highlight the efficacy of DBS in improving seizure control in a substantial proportion of patients, underscoring its therapeutic value in managing drug-resistant epilepsy [15-19].

Side effects: The overall safety profile of DBS for drug-resistant epilepsy varied across the studies, showing a generally low incidence of life-threatening complications such as symptomatic hemorrhage, infection, or sudden unexpected death in epilepsy (SUDEP). However, several non-life-threatening adverse events (AEs) were reported, with neuropsychiatric side effects such as depression, memory problems, and anxiety. Stimulation-induced adverse events, such as unintended seizures or movement disorders, were also reported in some patients.

**Figure 4:**
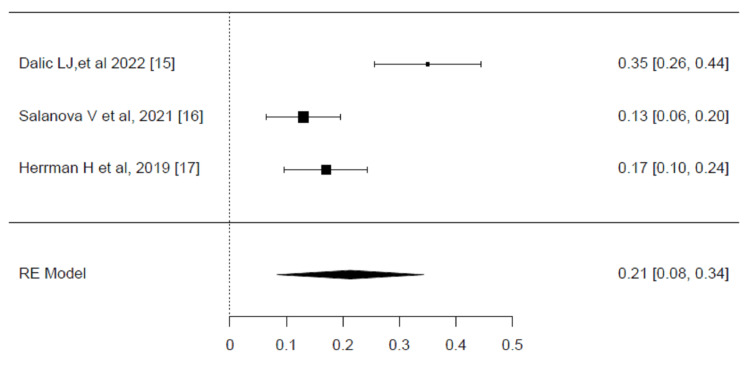
Meta-analysis of adverse events Forest plot showing the pooled effect sizes for adverse events (AE) among patients treated with deep brain stimulation (DBS). The pooled effect size was 0.21 (95% CI, 0.08–0.34), indicating a low incidence of AEs. Significant heterogeneity was detected (I² = 88.29%, p < 0.001), and the restricted maximum likelihood model was used for the analysis. Source: [15-17]

The AE results of DBS are presented in the forest plot (Figure 4). A significant degree of heterogeneity was detected across the studies, with an I² value of 88.29% (p<0.001). The pooled effect size for AEs in patients treated with DBS was statistically significant, with a value of 0.21 (95% CI, 0.08-0.34, P <0.001). This result indicates that the overall incidence of AEs was relatively low, but not negligible, with an estimated 21% of patients experiencing some form of adverse event. These findings emphasize the importance of balancing the benefits of seizure reduction with the potential for adverse effects in clinical decision-making [15,16,17].

**Figure 5:**
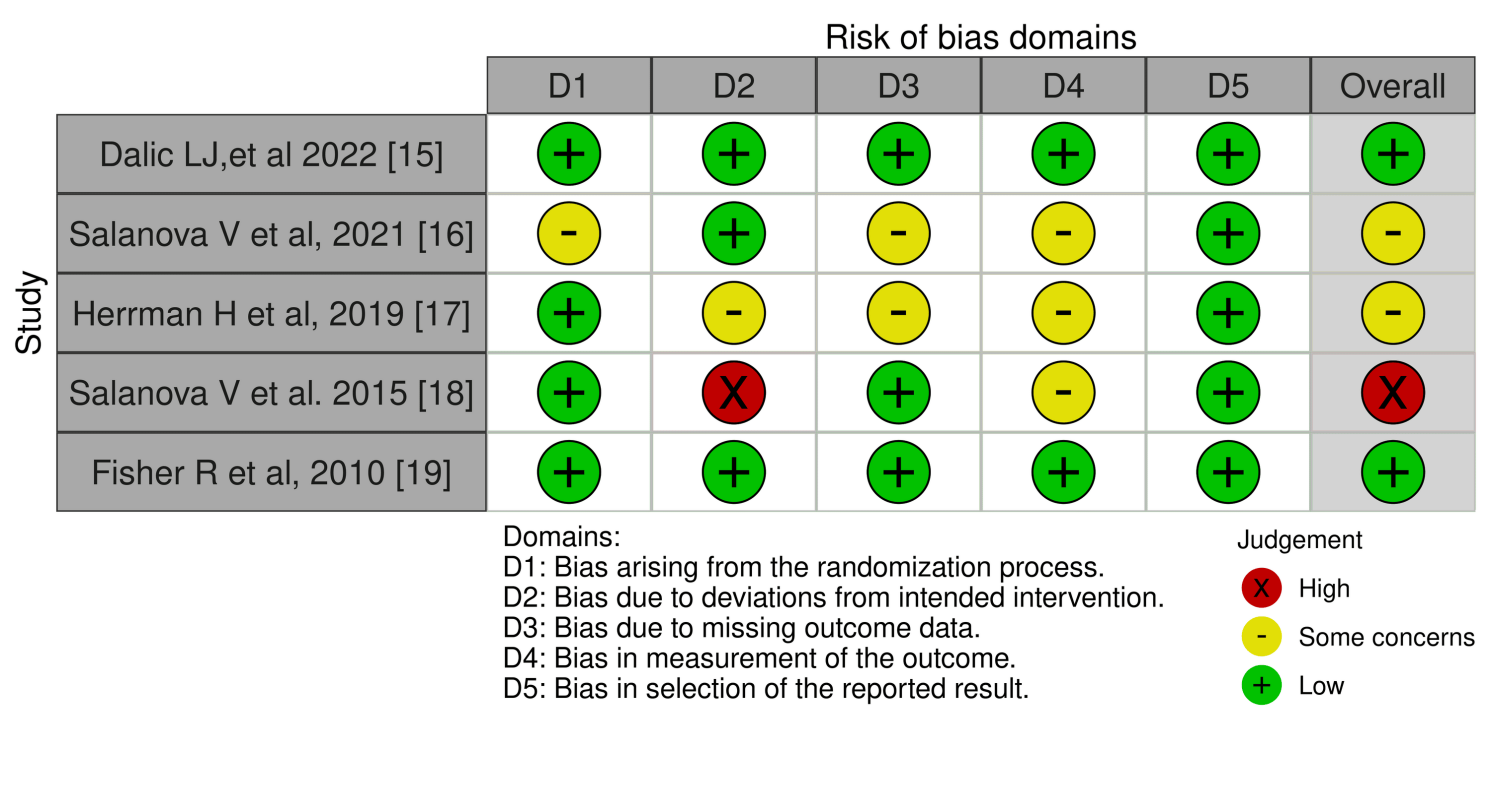
Risk-of-bias traffic light plot Source: [15-19]

**Figure 6:**
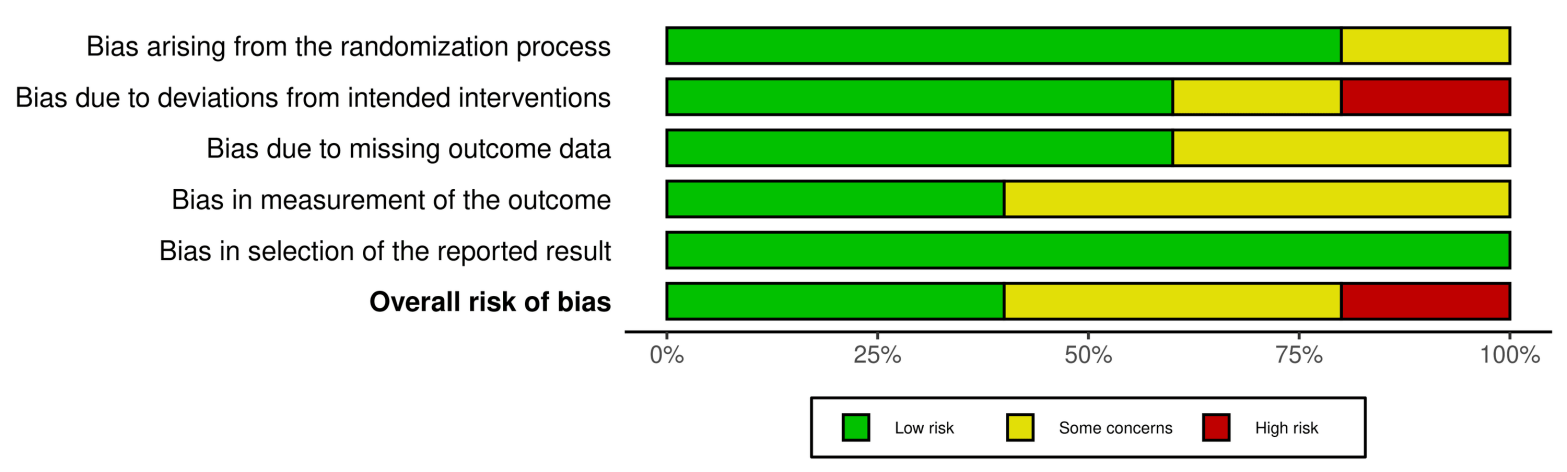
Risk-of-bias summary

Risk of bias assessment (ROB): ROB was assessed using the ROB2 tool for all included trials. The summary of the ROB in each domain as well as the overall risk is provided in Figures 5, 6. The ROB regarding randomization was low in four of the included studies [15,17-19]. Regarding the deviation from the intended intervention, one study showed high ROB [18]. In addition, two studies showed some concerns about missing outcome data [16,17]. The measurement of outcomes showed some concerns in three of the included studies [16-18]. The risk of selective reporting of outcomes was low in all trials (Figures 5, 6) [15-19].

Discussion

This systematic review and meta-analysis aimed to assess the efficacy and safety of DBS for managing DRE. The findings of this review concluded that there is a significant relationship between DBS and the improvements associated with the application of DBS among this specific population. The analysis of SR and RR from DBS studies showed that there was significant heterogeneity between trials. For seizure reduction, the pooled effect was statistically significant, with an effect size of 0.51 (P < 0.001), indicating a meaningful reduction in seizure frequency. Similarly, the RR was statistically significant, with a pooled effect size of 0.54 (P <0.001), showing a substantial proportion of patients achieving a ≥50% reduction in seizures.

Efficacy of DBS

The current review shows that the studies demonstrated that DBS significantly reduces seizure frequency, with significant long-term benefits reported. The SANTÉ trial by Salanova et al., 2021 [16] and earlier studies by Salanova et al., 2015 [18], show a median seizure reduction of 75% after 7 years, suggesting profound efficacy. Similarly, Fisher et al. supported these findings with a 56% median reduction in seizure frequency over two years and a substantial proportion of participants achieving a ≥50% reduction [19]. These outcomes align with the other studies in the literature as reported by several authors. For instance, in 2019, Zangiabadi et al. affirmed that DBS can be highly effective for DRE, especially in patients who are unresponsive to other treatments, though they showed variability in outcomes [20]. In addition, in 2023, Sobstyl et al. supported these results, showing that anterior thalamic DBS significantly reduces seizures in intractable cases, emphasizing its effectiveness [21]. However, variability in responder rates is acknowledged. As well, in 2021, Passamonti et al. and Guery and Rheims [9] affirmed the variability in efficacy, emphasizing that despite DBS being beneficial, the success rate can differ based on patient characteristics and stimulation parameters [22].

However, some studies, such as Herrman et al. (2019) [17], reported less improved outcomes compared with other studies, with a 22% reduction in seizure frequency after six months. This lower efficacy may be attributed to the differences in study design, patient demographics, and stimulation parameters.

Safety Profile

Only three trials reported AE with significant incidence among the included studies (P = 0.001) [15-17]. Neuropsychiatric outcomes remained a concern. The SANTÉ trial reported a low SUDEP rate of 2.0 per 1000 person-years, which is considered comparable to other neuromodulation treatments like vagus nerve stimulation (VNS) [18]. Fisher et al. also reported no symptomatic hemorrhages or brain infections, emphasizing the safety of DBS procedures [19]. Nevertheless, there have been increased reports of cognitive and mood-related issues in the literature. For instance, Zangiabadi et al. confirmed that DBS has a generally good safety profile but acknowledges potential neuropsychiatric effects such as mood changes and cognitive disturbances [20].

In addition, Sobstyl et al. reported mild adverse effects related to cognitive and mood changes [21], aligning with the neuropsychiatric concerns noted in the Fisher study [19]. In addition, Benedetti‑Isaac et al. found that despite DBS's efficacy in significantly reducing seizures, behavioral and mood changes should be considered with caution [23].

Responder Rates and Seizure Freedom

Variability in how well DBS works for different patients is a key consideration. While studies like the SANTÉ trial and earlier research showed high rates of response and some cases of complete seizure freedom, other studies, such as those by Herrman et al. (2019) [17] and Dalic et al. (2022) [15], observed less frequent seizure freedom, with only about half of the participants in the ESTEL trial experiencing a significant reduction in their seizures [15]. This was consistent with another study conducted by Larkin et al., 2016, which also noted this variability, highlighting that while DBS can be effective, the results can differ among individuals [24]. This underscores the need to identify factors that predict how well a patient might respond to DBS, helping select treatment and improve outcomes.

## Conclusions

In conclusion, the evidence supports DBS as a viable treatment option for DRE, with significant reductions in seizure frequency and a generally considerable safety profile. However, the variability in efficacy and responder rates across studies underscores the need for continued research to refine patient selection criteria, optimize stimulation parameters, and explore the differential effects of targeting various thalamic nuclei. Future studies should aim to identify biomarkers predictive of DBS response and better understand the neurophysiological mechanisms underlying its effects to enhance therapeutic efficacy and minimize adverse outcomes.
